# Impact of Feeding Frequency on Growth Performance and Antioxidant Capacity of *Litopenaeus vannamei* in Recirculating Aquaculture Systems

**DOI:** 10.3390/ani15020192

**Published:** 2025-01-13

**Authors:** Qinlang Liang, Gang Liu, Yazhi Luan, Jiangbo Niu, Yasong Li, Huwei Chen, Ying Liu, Songming Zhu

**Affiliations:** 1 College of Bio-Systems Engineering and Food Science, Zhejiang University, 866 Yuhangtang Road, Hangzhou 310058, China; liangql@tongwei.com (Q.L.);; 2 Dongying Tongwei Co., Ltd., Dongying 257092, China; luanyz01@tongwei.com (Y.L.);; 3 Ocean Academy, Zhejiang University, 866 Yuhangtang Road, Hangzhou 310058, China

**Keywords:** white shrimp, aquaculture, feeding frequency, automatic feeders, growth, antioxidant capacity

## Abstract

Feeding frequency is crucial for the growth and development of white shrimp (*Litopenaeus vannamei*) at various life stages. Although higher feeding frequencies can enhance growth, manual feeding methods significantly increase labor costs. Automatic feeding systems offer a cost-effective and efficient alternative, yet their application in intensive shrimp culture remains under-researched. This study evaluates different feeding frequencies for *L. vannamei* in intensive aquaculture tanks, focusing on growth performance, survivability, feed utilization, digestive and antioxidant capacities, and economic viability. Juvenile shrimp (3.85 ± 0.3 g) were cultured for 63 days with feeding frequencies of 6, 8, 10, and 12 times/day (A6, A8, A10, and A12 groups, respectively) using automatic feeders, with a control group fed manually 6 times/day (M6). Results indicated that automatic feeding significantly improved final body weight, specific growth rate, and feed conversion ratio compared to manual feeding. Among automatic feeding groups, A6 and A8 showed optimal performance, with a quadratic regression identifying 7.83 times/day as the optimal frequency. While digestive enzyme activity remained consistent across groups, A6 and A8 demonstrated significantly higher antioxidant enzyme levels (superoxide dismutase (SOD) and glutathione peroxidase (GPx)) and lower lipid peroxidation (MDA), suggesting superior digestive and antioxidant capacities. Pearson’s correlation confirmed a positive relationship between SOD and GPx. Economically, the A8 group achieved the highest profitability. Consequently, a feeding frequency of 6–8 times/day using automatic feeders is recommended as an optimal and feasible strategy for intensive white shrimp culture in this life stage.

## Simple Summary

This study investigated different feeding frequencies for white shrimp in intensive aquaculture using automatic feeders, and found that feeding 6–8 times/day resulted in higher growth, better feed conversion, improved digestive and antioxidant capacity, and higher profits compared to manual feeding. We believe that our study makes a significant contribution to the literature by demonstrating the effectiveness of automatic feeders and different feeding frequencies in white shrimp aquaculture. The findings highlight the benefits of feeding 6–8 times/day, leading to improved growth, feed utilization, and antioxidant capacity, with economic advantages for intensive shrimp culture operations.

## 1. Introduction

Feeding is a critical component of intensive shrimp aquaculture, accounting for approximately 60% of total production costs [1]. Effective feed management aims to optimize feeding practices by preventing overfeeding, minimizing waste, reducing cannibalism, and enhancing production efficiency [2]. Proper feeding strategies not only promote shrimp growth and production with high feed efficiency, but also minimize negative impacts on water quality [3]. In intensive shrimp farming, feeding management encompasses key elements such as feeding frequency, timing, ration, and the method of feed dispersal [3]. Feeding frequency is crucial to ensuring a good feeding method [4]. An optimal feeding frequency helps to improve shrimp growth and development, as well as enhances the feeding and feed utilization efficiency of shrimp. At the same time, it can reduce feed coefficient, heterogeneity, and environmental pollution, thereby improving total production and economic benefits. When shrimp are raised at a high density in industry shrimp culture, feeding management requires more effort as there is greater competition among *L. vannamei* that limits the yield of shrimp [5].

In the realm of crustaceans, *L. vannamei* exhibits a distinct physiological configuration where the hepatopancreas assumes the combined roles typically attributed to the intestine, liver, and pancreas in vertebrates. This unique organ plays a pivotal role in the shrimp’s digestive process and metabolic activities related to growth, as it is where the synthesis and secretion of digestive enzymes occur. The efficiency of these enzymes is thus a critical measure of the shrimp’s capacity for nutrient absorption and overall health. According to Vogt [6], the activity levels of digestive enzymes within the hepatopancreas are indicative of the organism’s digestive functionality and metabolic health, underscoring their significance in nutritional studies. It has been traditionally believed that increasing the frequency of daily feedings leads to accelerated shrimp growth, improved feed conversion efficiency, and enhanced water quality [7,8]. Xu, Xu, Su, Hu, Xu, Li, Wen, and Cao [9] found that daily feeding frequency can significantly impact the growth, feed utilization, digestive enzyme activity, and body composition of juvenile *L. vannamei* reared in biofloc-based, zero-exchange intensive systems. However, contrasting studies suggest that providing multiple feed rations may not offer additional benefits for the culture of *L. vannamei* and *Penaeus monodon* [10,11]. These findings highlight the need for a balanced approach to feeding frequency, depending on system conditions, growth stage, and species-specific requirements. These findings suggest the potential for optimizing feeding schedules to improve outcomes in shrimp aquaculture as well. Despite the importance of feeding frequency, data specific to shrimp, particularly *L. vannamei*, remain sparse. This study aims to fill this knowledge gap by investigating the effects of different feeding frequencies on shrimp growth and health in a simulated commercial setting, examining both automatic and manual feeding methods.

Automatic feeders are extensively employed in intensive aquaculture, often setting feeding rations and frequencies to maximize fast growth, even at the cost of using excess feed. However, this study emphasizes the importance of focusing feeding strategies on physiological needs rather than solely on rapid growth performance in aquafarming practices [12]. Therefore, feeding frequency should be further optimized, considering the physiological adaptability and welfare of aquatic species. Automation of feeding is essential in high-density, large-scale shrimp culture to overcome the time and labor constraints associated with manual feeding. Additionally, the key advantage of automatic feeders is their ability to enable frequent feeding, even during the night [9].

By conducting feeding trials under conditions that mirror those of commercial shrimp farms, this study assesses the impact of various feeding frequencies on key indicators of shrimp health and performance, including growth rates, digestive enzyme activity, antioxidant levels, and gene expression related to metabolism. The findings are intended to guide the application of automatic feeders in intensive shrimp farming, offering a scientific basis for enhancing shrimp production efficiency and sustainability.

## 2. Materials and Methods

### 2.1. Experimental Shrimp

**Figure 1:**
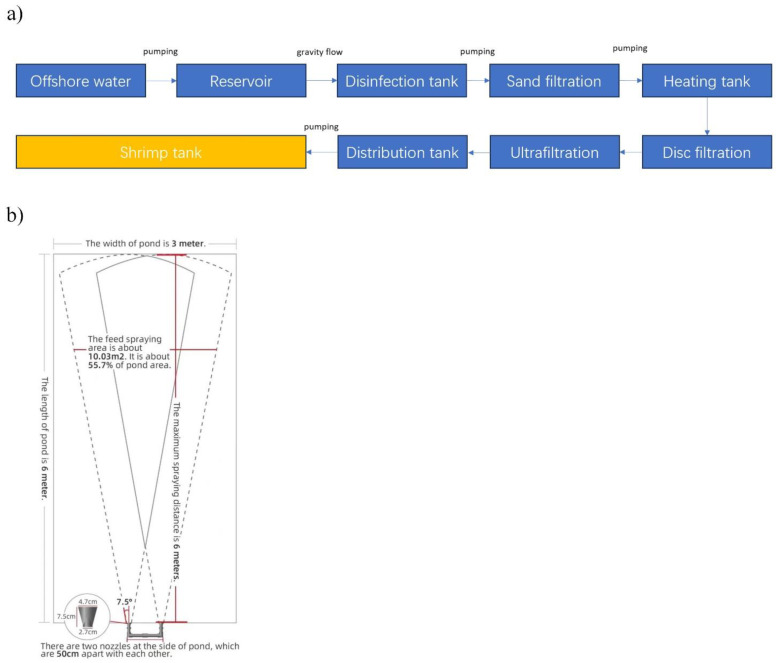
(**a**) The schematic design of the recirculating aquaculture system, (**b**) The feeding zone of the automatic feeding machine.

The experimental shrimp (*Litopenaeus vannamei*) were sourced from Tongwei Aquatic Technology Co., Ltd. (Dongying, Shandong, China). Upon reaching an average body weight of approximately 4 g, healthy shrimp (mean weight: 3.85 ± 0.03 g) were randomly selected and subjected to a 24 h fasting period. Subsequently, they were introduced into a controlled aquaculture system. The system consisted of an industry-standard tank with dimensions of 1.5 m in depth and a surface area of 18 m^2^. The shrimp were stocked at a density of 300 individuals per cubic meter, with the water depth maintained at 1 m. The schematic of the recirculating aquaculture system is shown in Figure 1a.

### 2.2. Experimental Design and Management

Based on previous production experience [13] and prior research [9], this study included six experimental groups: one manual feeding control group (M6) and five automatic feeding groups with different frequencies—12 times/day (A12), 10 times/day (A10), 8 times/day (A8), and 6 times/day (A6)—with the commercial feed (crude protein ≥ 43%, crude fat ≥ 6%, crude fiber ≤ 5%, crude ash ≤ 15%, total phosphorus ≥ 1.2%, lysine ≥ 2.6%, moisture ≤ 12%) for *L. vannamei* (Tongwei group, China). Each group was housed in 3e parallel tanks, totaling 18 tanks in the facility. A commercial automatic feeding machine from Huixintai Co., Ltd. (Dalian, China) was utilized, with its operational principle and feeding area illustrated in Figure 1b.

All experimental groups maintained consistent daily feeding amounts, water exchange rates, feeding methods, and other conditions, with feeding completed within 20 min, and the feeding quantity was recorded daily for the calculation of the feeding conversion ratio (FCR) and the feeding cost. The 63-day experiment period was sufficient to demonstrate growth performance due to the rapid growth rate of the shrimp species. Seawater was purified through sand filtration and UV sterilization. The experimental plant operated as a recirculating aquaculture system, generally eliminating the need for water changes to maintain water quality. Shrimp (*L. vannamei*) were weighed every 7 days, and daily feed amounts were recorded to calculate growth performance and yield and to perform the cost analysis.

### 2.3. Water Quality and Shrimp Sampling

During the experimental period, water temperature, dissolved oxygen (DO) levels, and pH were monitored daily using a multiparameter water quality instrument (FG4-FK, Mettler Toledo, Zurich, Switzerland). Water samples (100 mL) were collected before feeding and filtered through pre-dried and pre-weighed GF/C filter paper under vacuum pressure. The concentrations of total ammonia nitrogen (TAN), nitrite (NO_2_^−^-N), and nitrate (NO_3_^−^-N) in the filtrate were determined following the standard methods outlined by APHA (1998).

After 63 days of standardized cultivation, the *L. vannamei* were harvested and weighed, and the yield, specific growth rate (SGR), and feed conversion ratio (FCR) were recorded. Twenty shrimp from each group were randomly harvested and euthanized using thermal shock for final sampling, which included recording the average body weight and tissues.

The hepatopancreases were rinsed, dried, and collected in a 5 mL cryopreservation tube. Subsequently, the samples were frozen in liquid nitrogen and stored at a low temperature (−80 °C) for the determination of digestive enzyme and antioxidant enzyme activities.

### 2.4. Growth Performance

The growth performance was calculated using the recorded data according to the following equations:

Weight gain rate (WGR):

(1) $$WGR=\frac{W_{2}-W_{1}}{W_{1}}$$

Specific growth rate (SGR):

(2) $$SGR=\frac{{lnW}_{2}-{lnW}_{1}}{t_{2}-t_{1}}\times 100$$

Feed conversion ratio (FCR):

(3) $$FCR=\frac{feed\;supply\;(kg)}{shrimp\;biomass\;increase\;(kg)}$$

Survival (SUR):

(4) $$SUR=\frac{N_{2}-N_{1}}{N_{1}}$$

*W*_1_ and *W*_2_ are the weights at *t*_1_ (initial body weight) and *t*_2_ (final body weight). *N*_1_ = initial number of shrimps and *N*_2_ = final number of shrimps.

### 2.5. Determination of Digestive Enzyme and Antioxidant Enzymes Activities in the Hepatopancreas

The hepatopancreases were taken from a −80 °C refrigerator and thawed in a 4 °C refrigerator. The hepatopancreases in each group were pooled and placed in a 10 mL centrifuge tube. Physiological saline was added in a certain proportion, and the samples were thoroughly crushed with a tissue crusher (HUXI, FJ200-SH, Shanghai, China). Then, the crushed samples were divided into 1.5 mL enzyme-free centrifuge tubes and operated on ice. They were subsequently centrifuged at 4 °C for 10 min at 4500× *g*, and the supernatant was carefully collected and refrigerated for later use. The activity indicators of digestive enzymes, such as TP and amylase, including α and β-AMS in the intestine, were determined. Additionally, antioxidant enzyme activity indicators such as MDA, SOD, and GPx were tested following the kit’s instructions (Nanjing Jiancheng Biotechnology Research Institute, Nanjing, China).

### 2.6. Antioxidant Genes Expression

Quantification of gene expression was conducted using 15 samples per treatment, obtained by dividing the pools. Hepatopancreas samples were homogenized in TRIzol^®^ reagent (Invitrogen; Carlsbad, CA, USA) following the manufacturer’s instructions. RNA was quantified spectrophotometrically and evaluated for quality using agarose gel electrophoresis (1% *w*/*v*). Residual DNA was removed from 5 μg of total RNA using 1 U DNAse (Invitrogen) at 37 °C for 60 min, followed by inactivation with 15 mM EDTA at 75 °C for 10 min. For relative expression analysis, 1.0 μg of RNA from the experimental hepatopancreas was mixed with 0.5 μg oligo-dT, incubated at 70 °C for 5 min, then chilled on ice for 5 min. Retro transcription was performed using 1 μL of ImProm-II reverse transcriptase (Promega; Madison, WI, USA), 4 μL buffer 5 × ImProm-II, 30 mM MgCl_2_, 0.5 mM dNTP, and 40 U RNAsin (Promega) in a 20 μL reaction; incubated at 25 °C for 5 min; extended at 42 °C for 60 min; and inactivated at 70 °C for 15 min.

The expression of MnSOD and GPx genes was analyzed using a CFX96 real-time system (Bio-Rad Laboratories; Hercules, CA, USA). Each reaction consisted of 3 μL GoTaq Flexi Buffer, 2.5 mM MgCl_2_, 0.15 mM dNTPs, 0.45 U GoTaq Flexi DNA polymerase (Promega), and 1× EvaGreen DNA-binding dye (Biotium; Hayward, CA, USA) in a final volume of 15 μL The primer pairs and their concentrations for each gene are provided in Table 1. Reverse PCR (RT-qPCR) analysis was performed with an initial step at 94 °C for 3 min, followed by 40 cycles at 94 °C for 10 s and 60 °C for 30 s. A dissociation curve was generated in the range of 60 °C to 94 °C to confirm the specificity of the amplified products. All samples were analyzed in triplicate, and relative gene expression levels were calculated as previously described [14]. The RT-qPCR efficiency of each primer pair (Table 1) was determined by constructing standard curves using 10-fold cDNA dilutions ranging from 1:10 to 1:100,000. The efficiency of the PCR reaction (E) was calculated from the slope of the standard curve using the formula E = 10 (−1/slope) − 1. The Elongation Factor (EF1A) gene was used as an internal control to normalize the expression levels of the target genes.

### 2.7. Economic Analysis

The feeding cost was determined based on the recorded weight of shrimp feed, with a cost of CNY 8000 per ton. Energy expenses for water circulation and aeration were calculated using the readings from the electricity meter and divided by the corresponding tank production. Labor costs were estimated based on the workers’ salaries, with each worker earning CNY 4000 per month. Probiotic consumption was calculated by dividing the recorded amount of commercial probiotics used by the production output of the corresponding tank.

### 2.8. Data Analysis

Statistical analysis was conducted using IBM SPSS 20.0 for Windows (IBM Corporation, New York, NY, USA). Significance was determined at *p* < 0.05. One-way analysis of variance (ANOVA) was utilized to identify significant differences in growth parameters, digestive parameters, and antioxidant parameters. Tukey’s test at the *p* < 0.05 level of significance was used for post hoc analysis to determine group differences. The Pearson’s correlation among physiological enzymes, gene expression, and growth parameters was calculated and visualized using R 4.3.0. in the *corrplot* package 0.92 and *pheatmap* package.

## 3. Results and Discussion

### 3.1. Growth Performance of L. vannamei and Suitable Feeding Frequency

Throughout the duration of the experiment, we meticulously maintained the water quality within optimal parameters. The water temperature was held at an average of 29.33 ± 1.21 °C; salinity was measured at 27.21 ± 2.11 g L^−1^; DO levels were kept at 6.37 ± 0.39 mg L^−1^; pH levels were stable at 8.10 ± 0.15; and TAN, NO_2_-N, and NO_3_-N were controlled at 3.24 ± 1.20 mg L^−1^, 2.13 ± 0.85 mg L^−1^, and 11.20 ± 3.12 mg L^−1^, respectively.

Our observations revealed that groups A6 and A8 demonstrated superior FCR, as highlighted in Table 2, signifying more effective feed utilization within these groups. Conversely, increasing the feeding frequency to twelve times per day led to a noticeable rise in FCR, indicating a decrease in feed efficiency. In fact, under the experimental conditions (where feed had to be consumed within 20 min), higher feeding frequencies often disrupted the normal functioning of the digestive system [16,17]. This phenomenon corroborates findings from previous research [18,19,20], which suggest that an optimal feeding frequency can enhance feed conversion and growth metrics in aquatic species. The deterioration in feed utilization at higher feeding frequencies could be attributed to declining water quality, which adversely affects animal health and growth performance [21]. Furthermore, excessive feeding can lead to increased organic load and nutrient imbalances in the culture environment, exacerbating stress and susceptibility to disease in shrimp [22]. These insights underscore the importance of identifying and maintaining an optimal feeding regime to ensure sustainable and profitable shrimp farming practices.

This correlation underlines the importance of balancing feeding practices with water quality management to optimize growth and health in aquaculture operations [23]. During the shrimp grow-out phase, employing high feeding frequencies—“eating small, frequent meals”—became a standard practice to promote accelerated growth rates [9,17]. While this method did boost growth, it also significantly increased labor demands, highlighting the crucial need for technological solutions [21]. The introduction of automatic feeding machines emerged as a pivotal development in addressing this challenge [24]. These machines ensured a consistent and precise delivery of feed, which was instrumental in reducing labor requirements. By automating the feeding process, these devices not only maintained the necessary nutritional input for optimal shrimp growth, but also played a key role in enhancing operational efficiency [25]. The adoption of such technology represents a significant step forward in aquaculture, combining advancements in feeding strategies with innovations in automation to achieve both improved growth outcomes and labor savings. Moreover, automatic feeding systems contribute to better water quality management by preventing overfeeding and the associated increase in organic waste, which can degrade water conditions and harm shrimp health [26]. By integrating these technological solutions, aquaculture operations can achieve a more sustainable balance between high productivity and environmental stewardship. This approach not only supports the economic viability of shrimp farming but also promotes the long-term health of aquatic ecosystems, ensuring that both industry and environment can thrive together [27].

**Figure 2:**
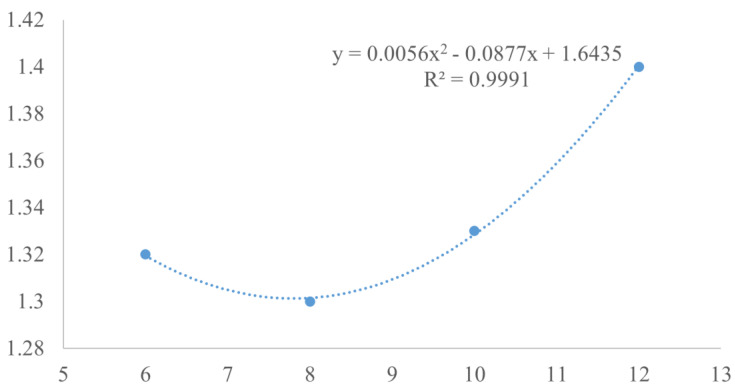
Relationship between FCR and feeding frequency of *L. vannamei* based on a quadratic polynomial regression analysis.

Table 2 reveals that the FBW was consistently higher across all groups utilizing automatic feeders. Notably, within these automated feeding regimens, the FBW in the A8 and A10 groups significantly surpassed that of the M6 group. The FCR peaked in the M6 and A12 groups, followed by a lesser but still notable increase in the A10 group. Conversely, the A6 and A8 groups exhibited no significant difference in FCR (*p* > 0.05), suggesting an optimal feeding frequency between 6 and 8 times per day. This hypothesis is further supported by the quadratic regression equation of FCR, which indicates an ideal feeding frequency of 7.83 times/day (Figure 2), implying that the A8 group may achieve the most favorable outcomes. Xu et al. [8] also found that a higher feeding frequency was suitable for the *L. vannamei*. While a higher feeding frequency can escalate labor costs, an insufficient feeding frequency may compromise feed efficiency and impede growth performance.

Extending this analysis, it is essential to consider the implications of automated feeding systems on overall operational efficiency. Automation not only enhances precision in feed delivery but also reduces human error, leading to more consistent growth rates and potentially lower feed wastage [28,29]. Future studies should investigate the long-term economic impacts of varying feeding frequencies, taking into account both feed costs and labor expenses. Additionally, examining the behavioral responses of animals to different feeding schedules could provide insights into optimizing welfare alongside performance metrics [30]. Integrating advanced technologies, such as real-time monitoring and adaptive feeding systems, could further refine these practices, ensuring both economic and ethical benefits in shrimp management.

The weekly WGR from Table 3 demonstrates that the A8 group achieved the highest WGR among all treatments, significantly outperforming the M6 group (*p* < 0.05). By the 63rd day, the A12 group exhibited a significantly lower WGR within the automatic feeding groups (*p* < 0.05), indicating that this frequency may be suboptimal for shrimp rearing. At the experimental endpoint, the A8 group recorded the highest FBW (18.45 ± 2.56), although there was no significant difference in FBW among the automatic feeding groups (A6, A8, and A10) at this stage (*p* > 0.05). Additionally, the A8 group reported the lowest FCR (1.30 ± 0.08), alongside the highest survival rate (98.48 ± 1.35%) and yield (108.75 ± 7.32 kg tank^−1^) (Table 2).

These findings suggest that initial differences in growth among shrimp subjected to various feeding frequencies were not pronounced in early stage of the experiment (Table 3). However, as the experiment progressed beyond three weeks, these differences became more evident, underscoring the importance of feeding frequency as a critical determinant in sustainable shrimp production over the long term. Moreover, the results highlight the efficiency of automatic feeders in maintaining optimal feeding schedules, thereby enhancing growth performance and operational efficiency.

### 3.2. Effect of Feeding Frequency on the Digestive Enzymes in the Hepatopancreas

**Figure 3:**
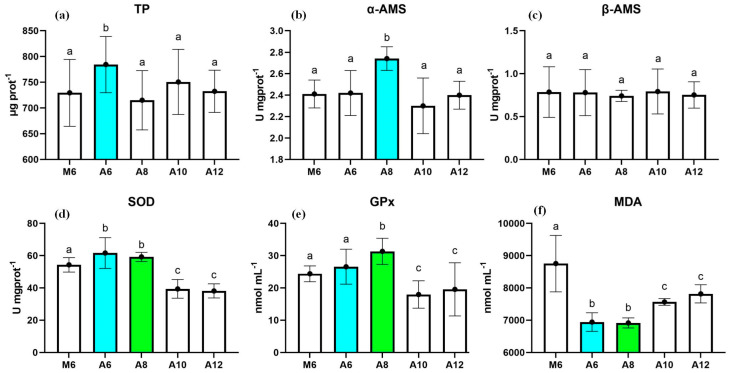
The activities of total protein (TP) (**a**), α a, β-amylase (**b**,**c**), superoxide dismutase (SOD) (**d**), glutathione peroxidase (GPx) (**e**), and malondialdehyde (MDA) (**f**) in *L. vannamei* cultured in five treatments at the end of the 63-day experiment. (*n* = 3). Different letters indicate significant differences (*p* < 0.05).

In our investigation, we quantified the activity of three key digestive enzymes—total protease (TP), α-amylase (α-AMS), and β-amylase (β-AMS)—within the hepatopancreas at the conclusion of the experimental period, as illustrated in Figure 3a–c. Our findings reveal that TP activity was notably enhanced in the A6 group, suggesting an elevated protein digestion efficiency under this feeding regime. Conversely, α-AMS activity reached its peak in the A8 group, indicating superior carbohydrate metabolism at this particular feeding frequency. Although β-AMS activity demonstrated variability across the treatments, it did not achieve statistical significance, pointing to a complex interaction between feeding frequency and enzyme activity that merits further exploration. The observed variations in enzyme activity align with previous research [31,32,33] which collectively suggests that tailored feeding regimens can markedly influence the digestive efficiency and, by extension, the growth performance of *L. vannamei* and other aquacultural species. These studies corroborate the premise that an optimal feeding frequency can enhance the digestive system’s functionality, leading to improved nutrient utilization and growth metrics.

This research underscores the critical impact of feeding frequency on the activity of digestive enzymes in *L. vannamei*, establishing a direct correlation with the shrimp’s growth performance. By optimizing feeding frequencies, we can significantly enhance the digestive capacity of *L. vannamei*, thereby promoting better growth outcomes and contributing to more efficient and sustainable aquaculture practices. Improved digestive capacity consistently corresponded to better yield [1].

### 3.3. Effect of Feeding Frequency on the Antioxidant Enzymes and Relative Genes Expression in the Hepatopancreas

In our study spanning 63 days, we meticulously measured the activities of SOD and glutathione peroxidase GPx within the hepatopancreas of the *L. vannamei*, particularly focusing on those in the A8 feeding group. The results, depicted in Figure 3d,e, showed a significant elevation in the activities of these antioxidant enzymes. Elevated levels of SOD and GPx are generally interpreted as indicators of an enhanced antioxidative defense mechanism within the organism, suggesting a superior capacity to neutralize oxidative stress.

The relationship between antioxidant enzyme activities and the overall antioxidative capacity of an organism has been extensively documented in the literature, particularly in studies focusing on dietary interventions, including the administration of probiotics and other nutritional supplements [32,34,35]. These investigations have consistently reported increases in SOD enzyme levels, underscoring the link between dietary composition and oxidative stress management in aquatic species.

Furthermore, research indicates that organisms, when faced with environmental stressors such as low salinity, engage in adaptive biochemical responses that increase the production of SOD, thereby enhancing their resilience and health by bolstering their antioxidative defenses [36,37]. Such adaptations are crucial for mitigating the adverse effects of reactive oxygen species (ROS) and preventing cellular and tissue damage. Our findings align with the hypothesis that higher activities of antioxidative enzymes like SOD and GPx reflect a more robust antioxidative capability within the group. This observation not only corroborates existing research but also enriches our understanding of the dynamic interplay between feeding strategies and the antioxidative defense mechanisms in *L. vannamei*. It underscores the potential of nutritional strategies in enhancing the health and stress resilience of aquaculture species, suggesting that optimizing feeding regimens can significantly contribute to the welfare and productivity of shrimp farming operations.

The A8 group presented a noteworthy finding with a significant reduction in MDA levels (Figure 3f), a primary indicator of lipid peroxidation. This reduction signals a decrease in oxidative stress, affirming the efficacy of this specific feeding frequency in enhancing the antioxidant defenses of the shrimp. Echoing our findings, research conducted by Muttharasi, Gayathri, Muralisankar, Mohan, Uthayakumar, Radhakrishnan, Kumar, and Palanisamy [38] also demonstrated a reduction in MDA levels among groups receiving diets enriched with antioxidants, specifically *Amphiroa fragilissima* crude polysaccharides encapsulated in *Artemia nauplii*. These parallel observations reinforce the premise that an optimized feeding frequency, such as 8 times per day, plays a pivotal role in bolstering the antioxidant capacity of *L. vannamei*, consequently diminishing the levels of oxidative stress.

This connection between feeding frequency and reduced oxidative stress is significant for aquaculture practices, suggesting that dietary management can serve as a critical tool in promoting the health and resilience of shrimp populations [39]. By mitigating oxidative damage through enhanced antioxidant defense mechanisms, such feeding strategies offer a promising approach to improving the overall well-being and productivity of *L. vannamei* in controlled farming environments. The implications of these findings extend beyond immediate health benefits, potentially contributing to the sustainability and efficiency of aquaculture operations by supporting the cultivation of shrimp that are better equipped to withstand environmental stresses.

Our investigation into the regulatory mechanisms of antioxidant defenses in *L. vannamei* illuminates the critical role of gene expression in orchestrating the production and activity of antioxidant enzymes. The expression levels of specific antioxidant genes serve as a barometer for the presence and potency of antioxidant enzymes within the organism [13]. Supporting this notion, research conducted by Liu, Ye, Liu, and Zhu [1] has previously established a positive correlation between the augmentation of SOD levels and the dietary inclusion of commercial probiotics, highlighting the impact of nutrition on gene expression and, consequently, antioxidant defense mechanisms.

**Figure 4:**
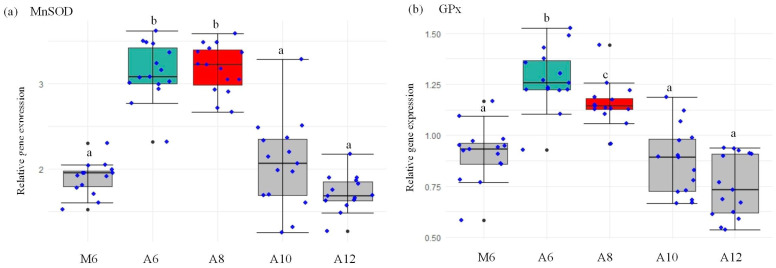
Gene expression levels of MnSOD (**a**) and GPx (**b**) in the hepatopancreas of *L. vannamei* in the five treatments. Data are the sample values and standard deviations. Different lowercase letters mean significant differences (*p* < 0.05). Different colors highlight the significantly different groups.

In our comprehensive analysis, we observed a marked elevation in MnSOD gene expression within the A6 and A8 groups (Figure 4a), signifying a statistically significant increase in SOD enzyme levels. This elevation in gene expression directly correlates with an amplification of enzyme activity, underscoring the relationship between genetic regulation and functional enzyme dynamics. Such an increase affirms the premise that heightened gene expression facilitates enhanced antioxidant enzyme synthesis [40]. Furthering our exploration, the conclusion of the 63-day feeding trial revealed a pronounced increase in GPx transcript levels in the A6 group (Figure 4b). This finding is particularly noteworthy given the indispensable role of the glutathione system in sustaining cellular redox equilibrium and executing detoxification processes. GPx, in concert with SOD, is instrumental in converting hydrogen peroxide (H_2_O_2_) to water, thereby mitigating oxidative stress through the efficient removal of free radicals [41].

The observed upregulation of MnSOD and GPx gene transcripts in the A6 and A8 groups not only underscores the enhanced antioxidative capabilities conferred by these feeding strategies, but also aligns with the documented shifts in enzyme activity levels. This congruence between gene expression patterns and enzymatic activity provides compelling evidence of the dynamic nature of antioxidant defenses, influenced by dietary and environmental factors [22]. Consequently, our findings highlight the significance of targeted nutritional interventions in promoting antioxidant capacity and safeguarding the health and vitality of aquaculture species.

**Figure 5:**
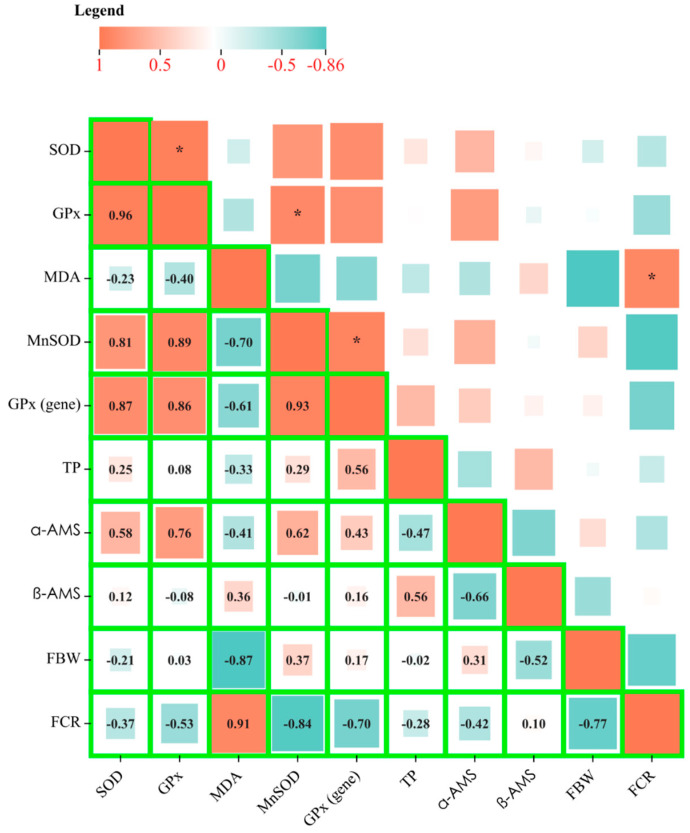
The correlation heatmap characterizes the Pearson correlation coefficients (r) between the physiological parameters and growth parameters. The orange color means the positive correlation, while the green color means the negative correlation. The depth of color indicates the size of the relationship. The asterisk (*) indicates a significant correlation (*p* < 0.05). Moderate correlation was defined following (0.5 < |r| < 0.8) and high correlation following (0.8 < |r| < 1), respectively.

Pearson’s correlation analysis, depicted in Figure 5, elucidates the synergistic interaction between SOD and its MnSOD, alongside GPx and its corresponding transcript levels, showcasing a positive correlation that underscores their collaborative impact on enhancing antioxidant defense mechanisms. This finding corroborates the notion that SOD and GPx, operating in concert, are pivotal to the organism’s antioxidant capacity, a dynamic thoroughly explored in preceding discussions.

Intriguingly, the analysis revealed that MDA levels inversely correlate with both SOD and GPx activities (Figure 5), suggesting that the pathways regulating MDA, a marker of lipid peroxidation, diverge from those governing SOD and GPx. This distinction underscores the complexity of the antioxidant system and highlights the unique role of MDA as an indicator of oxidative stress. Furthermore, a positive correlation between FBW and the activities of SOD and GPx was observed, implying that an enhanced antioxidant system is conducive to improved growth rates in shrimp. This association aligns with previous research [1,13], which has documented the beneficial impact of robust antioxidant defenses on the growth performance of aquatic organisms.

Taken together, these insights reveal a comprehensive enhancement in both digestive and antioxidant functions within the hepatopancreases of shrimp from the A8 group. This enhancement is evidenced by the decreased levels of MDA and the elevated activities and expression levels of SOD, MnSOD, and GPx. The collective findings underscore the integral role of optimized feeding regimens in promoting health and growth in *L. vannamei* through mechanisms that bolster the organism’s antioxidant capacity and mitigate oxidative stress.

### 3.4. Economic Analysis

In assessing the economic aspects of various feeding strategies for *L. vannamei* cultivation, our analysis meticulously accounted for diverse cost factors, including manual labor, energy for water exchange and aeration, as well as food and probiotic consumption. These expenses were then calculated relative to the total yield of shrimp to determine the average production cost, as detailed in Table 4. Our findings reveal that neither the highest feeding frequency nor manual feeding methods inherently guarantee the most cost-efficient approach to shrimp farming. Particularly within the recirculating aquaculture system (RAS), despite the necessity for periodic water changes or offset evaporation, the energy costs associated with water management represent a significant expense over a long time. Notably, the A8 group emerged as the most cost-effective among all evaluated groups, attributed largely to its superior yield efficiency. This enhanced cost-efficiency in the A8 group was further accentuated by comparing other operational costs, where the savings became even more pronounced due to the higher yield achieved.

The analysis also underscored that feed expenses constituted the largest proportion of total costs, accounting for approximately 60% of the overall expenditure, followed closely by energy costs. The latter is particularly salient in RAS setups, where the energy consumption for maintaining water quality and system operations is substantial [42]. Manual feeding, on the other hand, was identified as a time-intensive and laborious process that not only incurs higher costs but also tends to deliver less satisfactory outcomes in terms of shrimp growth and overall productivity.

Based on these insights, our study advocates for the adoption of automatic feeders in intensive shrimp aquaculture practices. Specifically, a feeding frequency of eight times per day was pinpointed as the optimal regimen, balancing cost-efficiency with effective resource utilization to maximize yield. This strategic approach underscores the potential of technological interventions in enhancing the economic viability and sustainability of *L. vannamei* farming.

## 4. Conclusions

This study highlights the importance of feeding frequency in optimizing the growth, health, and economic efficiency of *L. vannamei* in intensive aquaculture. Automatic feeding at 6–8 times per day significantly improved growth performance, feed conversion, and antioxidant capacity. The A8 group showed the best results, with the highest final body weight, lowest feed conversion ratio, and highest survival rate. Feeding frequency also impacted digestive enzyme activity, with A8 showing better protein and carbohydrate digestion. Additionally, A8 shrimp exhibited higher antioxidant enzyme (SOD and GPx) activities, indicating improved stress resilience. Economically, the A8 group was the most cost-effective, combining high yield with reduced labor and feed waste. In conclusion, automatic feeding systems with a frequency of eight times per day optimize shrimp growth, health, and operational efficiency. This strategy offers a sustainable and profitable solution for intensive *L. vannamei* farming, enhancing both productivity and environmental sustainability.

## Figures and Tables

**Table 1:** Primer pairs and the concentration for each qPCR testing.

Gene	Primer	Concentration (μM)	Efficency (%)	
EF1A	5′-ATGGTTGTCAACTTTGCCCC-3′	120	92.13	Sittikankaew, K., Hiransuchalert, R., Yocawibun, P., Yamano, K., Klinbunga, S. [13]
	5′-TTGACCTCCTTGATCACACC-3′			
MnSOD	5′-TCATGCTTTGCCACCTCTC-3′	90	95.62	Ji, P.-F.; Yao, C.-L.; Wang, Z.-Y. [14]
	5′-CCGCTTCAACCAACTTCTTC-3′			
GPx	5′-GAAGACCCGGTGACCCAAAA-3′	80	91.43	Guzmán-Villanueva, L.T.; Escobedo-Fregoso, C.; Barajas-Sandoval, D.R.; Gomez-Gil, B.; Peña-Rodríguez, A.; Martínez-Diaz, S.F.; Balcázar, J.L.; Quiroz-Guzmán, E. [15]
	5′-TTCTGTTTCTCCGCTCTCCG-3′			

**Table 2:** Growth performance of *L. vannamei* in the 63-day feeding trial.

	Treatments				
	M6	A6	A8	A10	A12
IBW (g)	3.85 ± 2.43 ^a^	3.89 ± 1.85 ^a^	3.82 ± 2.35 ^a^	3.85 ± 1.89 ^a^	3.83 ± 2.10 ^a^
FBW (g)	16.9 ± 2.93 ^a^	18.38± 2.39 ^b^	18.48 ± 2.56 ^b^	17.89 ± 3.09 ^b^	17.21 ± 2.33 ^a^
SGR (%)	11.82 ± 1.25 ^a^	12.62 ± 1.43 ^b^	12.98 ± 1.13 ^b^	12.81 ± 1.88 ^b^	12.80 ± 1.17 ^b^
FCR	1.43 ± 0.01 ^a^	1.32 ± 0.05 ^b^	1.30 ± 0.08 ^b^	1.33 ± 0.02 ^b^	1.40 ± 0.03 ^a^
SUR (%)	96.40 ± 1.40 ^a^	98.42 ± 0.31 ^b^	98.48 ± 1.35 ^b^	96.53 ± 1.33 ^a^	95.4 ± 1.25 ^a^
Yield (kg tank^−1^)	98.33 ± 5.63 ^a^	105.66 ± 6.42 ^b^	108.75 ± 7.32 ^b^	105.92 ± 4.21 ^b^	103.6 ± 5.21 ^b^

Values presented as means ± standard deviation. Different superscripts mean significant differences (*p* < 0.05) (*n* = 3). IBW, initial body weight; FBW, final body weight; SGR, specific growth rate; FCR, feed conversion ratio; SUR, survival.

**Table 3:** Body weight gain rate (WGR) of *L. vannamei* at each stage of the weekly growth cycle.

Days	Treatments				
	M6	A6	A8	A10	A12
7	25.97 ± 1.25 ^a^	25.96 ± 2.13 ^a^	28.79 ± 1.54 ^b^	28.61 ± 2.12 ^b^	28.72 ± 1.88 ^b^
14	25.97 ± 2.22 ^a^	25.10 ± 1.88 ^a^	25.01 ± 1.76 ^a^	25.04 ± 1.91 ^a^	24.14 ± 1.45 ^a^
21	16.20 ± 2.31 ^a^	17.13 ± 2.32 ^b^	17.24 ± 1.21 ^b^	17.92 ± 2.10 ^b^	16.34 ± 1.25 ^a^
28	15.49 ± 1.12 ^a^	17.27 ± 1.33 ^b^	17.05 ± 2.09 ^b^	16.88 ± 1.83 ^b^	16.71 ± 2.14 ^b^
35	16.21 ± 2.11 ^a^	17.34 ± 0.98 ^b^	17.18 ± 2.10 ^b^	17.76 ± 1.76 ^b^	16.85 ± 2.08 ^a^
42	16.05 ± 1.26 ^a^	17.00 ± 1.77 ^b^	16.77 ± 2.18 ^b^	16.68 ± 2.31 ^b^	15.55 ± 1.43 ^a^
49	15.91 ± 1.44 ^a^	17.13 ± 2.31 ^b^	17.94 ± 2.08 ^b^	17.22 ± 2.06 ^b^	14.79 ± 2.01 ^c^
56	15.36 ± 1.20 ^a^	17.87 ± 1.41 ^b^	18.75 ± 2.43 ^c^	17.84 ± 2.13 ^b^	15.30 ± 2.88 ^a^
63	14.27 ± 3.12 ^a^	15.16 ± 1.01 ^b^	16.23 ± 2.41 ^c^	15.22 ± 2.11 ^b^	14.89 ±1.31 ^a^

Values presented as means ± standard deviation. Different superscripts mean significant differences (*p* < 0.05) (*n* = 3).

**Table 4:** Average cost per kilogram of yield for *L. vannamei* cultured in the five treatments during the 63-day feeding trial.

	Treatments				
	M6	A6	A8	A10	A12
Manual work (CNY per kg^−1^ shrimp)	4.07	3.79	3.68	3.78	3.86
Energy for water exchange (CNY per kg^−1^ shrimp)	1.69	1.58	1.53	1.57	1.61
Energy for aeration (CNY per kg^−1^ shrimp)	1.71	1.59	1.54	1.59	1.62
Food consumption (CNY per kg^−1^ shrimp)	11.20	10.72	10.56	11.02	11.74
Probiotic consumption (CNY per kg^−1^ shrimp)	2.03	1.89	1.84	1.89	1.93
Total cost (CNY per kg^−1^ shrimp)	20.70	19.57	19.15	19.85	20.76
Total cost (USD per kg^−1^ shrimp)	2.89	2.73	2.67	2.77	2.89

(CNY = Chinese currency unit; USD 1 = CNY 7.1663).

## Data Availability

Data are unavailable due to privacy or ethical restrictions.

## Abbreviations

Final body weight (FBW); specific growth rate (SGR); lower feed conversion ratio (FCR); total protease (TP); malondialdehyde (MDA); superoxide dismutase (SOD); and glutathione peroxidase (GPx).
